# Environmental therapy: interface design strategies for color graphics to assist navigational tasks in patients with visuospatial disorders through an analytic hierarchy process based on CIE color perception

**DOI:** 10.3389/fpsyg.2024.1348023

**Published:** 2024-10-28

**Authors:** Weicong Li, Shangbing Ma, Yueling Liu, Haopai Lin, Huabin Lv, Wenwen Shi, Jinghui Ao

**Affiliations:** ^1^Faculty of Built Environment and Surveying, Universiti Teknologi Malaysia, Johor Bahru, Malaysia; ^2^School of Creativity and Design, Guangzhou Huashang College, Guangzhou, China; ^3^School of Art and Design, Guangdong University of Finance and Economics, Guangzhou, China; ^4^School of Culture Communication & Design, Zhejiang University of Finance and Economics Dongfang College, Haining, China

**Keywords:** visual–spatial barriers, spatial orientation, color and graphics, hierarchical analysis, interface design, environmental design, CIE 1976, spatial intervention

## Abstract

**Introduction:**

Environmental therapy theory has been applied in the research of disease prevention, and the effectiveness of using color and graphic designs to assist patients with spatial orientation has been confirmed. Visual-spatial impairments are common symptoms associated with cognitive decline. However, the interaction and driving factors between these impairments and spatial color and graphic designs remain unclear.

**Methods:**

This paper first discusses the correlation between the characteristics of visual-spatial impairments and environmental factors and then investigates the color preferences of such patients based on the CIE 1976 color system and the Analytic Hierarchy Process (AHP). Subsequently, the paper explores spatial design strategies conducive to spatial orientation from the perspective of adaptability to pathological characteristics, utilizing case study analysis.

**Results:**

(1) Pathological characteristics of visual-spatial impairments (such as difficulties in spatial orientation and spatial neglect) are related to environmental factors; (2) Emotional attachment factors play a key role in patients’ perception of satisfaction with environmental colors; (3) Color associations have the potential to strengthen spatial memory. Additionally, interface designs with high luminance, low saturation, and clear color differentiation facilitate patients’ recognition of space.

**Discussion:**

This paper posits that spatial interface design is a feasible approach to assist with spatial orientation, and it achieves this through a mediating process that progresses from influencing visual stimuli to cognitive memory and then to behavioral orientation. The article provides insights into the operational feasibility of this method.

## 1 Introduction

Rehabilitative spaces are defined as environments specifically designed to support patient rehabilitation, aiming to enhance recovery quality through adjustments in the external environment (Tijsen et al., 2019). The core concept of environmental intervention lies in optimizing the physical environment to boost rehabilitation outcomes (Willatt, 2011; Li et al., 2023), such as the high-level communication spaces created by radial layouts which benefit cognitive functions (Real et al., 2017). Effective spatial configurations can aid patient rehabilitation but require personalized adjustments based on individual pathological characteristics. Overall, the content of environmental therapy has shown a shift in focus from experience-based approaches to meeting specific needs, perceiving stimuli, and ultimately cognitive rehabilitation, alongside a deepening research trajectory from visual perception to perceptual psychology, human perception, behavioral medicine, and neuroscience (Li et al., 2024). The discussion on “environmental facilitation of rehabilitation” has now reached the levels of spirituality and quality, with ongoing debate over whether outcomes are primarily determined by the “individual” or the “environment,” a hot topic in academia. Proponents of active therapy (such as rehabilitation trainers and horticultural therapists) believe that practical labor can effectively enhance a patient’s physical functions and psychological self-efficacy (Erbino et al., 2015). Experts focused on participatory therapies (such as spatial designers and sensory therapists) tend to believe in the healing nature of spaces themselves, with patients benefiting from environments close to nature (Dinu Roman Szabo et al., 2023).

In fact, this paper posits that the maximization of environmental therapy benefits is achieved through the interaction between the individual and the environment. Given the complexity of the pathogenesis of visual impairments, a single-disciplinary perspective is insufficient to propose effective spatial design solutions comprehensively. Preliminary research has already demonstrated that spatial configurations significantly impact spatial orientation abilities. This study underscores the potential value of interface color and graphic design in enhancing spatial orientation abilities.

Visuospatial disorders reflect cognitive impairments in processing spatial information, typically involving the ability to perceive spatial orientation, distance, and depth (Derbie et al., 2022; Funk et al., 2013). Aging correlates positively with cognitive decline and exacerbates spatial orientation disorders (Courtes et al., 2019). This functional impairment manifests in diminished abilities to discern the shape of objects, proximity, and distance (Jafri et al., 2014), significantly affecting individuals’ capacity for independent living (Costa et al., 2020). A cognitive experiment on wandering behaviors in dementia patients confirmed the supportive role of behavioral guidance in spatial recognition (Weicong and Zhaoming, 2020). Indeed, the concept of environmental therapy was mentioned as early as Solomon (1943) nursing notes, and clinical data from Ulrich (1983) confirmed the substantial potential of environmental facilitation in rehabilitation. His proposed “Stress Recovery Theory” and Kaplan and Kaplan (2011) “Attention Restoration Theory (ART)” further demonstrated the positive impacts of natural environments on human visual attention and physiological states. Their research unveiled dual implementation pathways for environmental interventions, between ‘individual’ and ‘environment’.

At the individual level, studies suggest that enhancing cognitive abilities is fundamental to strengthening patients’ spatial orientation skills. Fricke et al. (2021) advocated incorporating spatial orientation training into routine tasks at care institutions, which not only reduces sedentary behaviors but also benefits cognitive functions. They organized a 12-week spatial cognition program (twice weekly) for 40 patients in Germany, finding that simple directional tests could improve orientation abilities, though more complex games like Floor plan bingo might not always be applicable. Compared to the landmark placement experiments of Coughlan et al. (2018) and Li et al. (2024) focused more on enhancing patients’ recognition of other spatial attributes (scale, structure, order). The literature also highlights cognitive training through cognitive maps (Pouyan et al., 2024), drawing (Kim and Chun, 2014), clock tests (Souillard-Mandar et al., 2016), object restoration (Brooker et al., 2019), and route experiments (Muffato and De Beni, 2020), aiming to strengthen mental structuring of objects. Contrasting these cognitive games, Delahaye et al. (2015) paid more attention to the translation of cognitive tasks into three-dimensional spaces. They introduced virtual maze training to test patients’ path differentiation and memory capabilities, finding that mild stress induced by the scenarios helped to stimulate cognitive flexibility.

At the environmental level, scholars advocate for creating spaces that assist these patients in spatial orientation. Li et al. (2024) noted that patients with visuospatial disorders struggle to accurately judge the direction of horizontal and vertical lines (Harris and Mander, 2014; Odin et al., 2018). They argued that rectilinear spatial forms, symmetrical wall designs, and orderly ground paving all benefit patients in determining their positions relative to spatial elements. From a cognitive perspective, Hu et al. (2021) found a significant relationship between gaze duration and visual complexity in an eye-tracking study with 16 patients. Participants demonstrated sensitivity to the cognitive factors of color, texture, and visual complexity, but were insensitive to shape factors. Based on this, Li et al. (2024) believed that reducing scene complexity could alleviate the difficulty of spatial orientation tasks and benefit the maintenance of walking posture. From a narrative perspective, Pouyan et al. (2021) emphasized the design of transitional spaces for the elderly’s potential in direction identification. They noted that systematic designs of hospital signage and color can enhance spatial continuity and aid elderly patients in navigation. Based on color association methods, Jamshidi et al. (2020) highlighted the role of distinctly warm and cool color designs in deepening personal impressions of space, thereby correlating color impressions with spatial functions. The process of information substitution can be controlled through spatial attributes, such as the color environment. Modern hospitals have emphasized the role of color in guiding visiting patients and family members, using various colors and signs to direct patients to their destinations (Chong et al., 2023). Utilizing color’s warning function, different color labels assigned to different medicine bottle surfaces can also control the occurrence of patient medication misuse at the source (Nayak et al., 2022).

Oriented towards behavioral change, recent research on spatial configuration aiding spatial orientation has primarily focused on ‘spatial color cognition’ (Calori and Vanden-Eynden, 2015; Kalantari et al., 2022; Jamshidi et al., 2020) and ‘visual path mapping’ (Natapov et al., 2020; Yang et al., 2011; Hidayetoglu et al., 2012). The former mainly explores the ontological issues of color and perception, aiming to answer how specific colors influence the construction of cognitive maps. The latter focuses on the role of visual elements in guidance, largely overlooking other sensory inputs. Those bridging medical and design studies with evidence-based design theories attempt to link ‘behavioral perception’ with ‘spatial cognition’, seeking from behavioral psychology and neuroscience how colors and shapes influence neural pathway activations, thus comprehensively explaining the impact of spatial configurations on orientation abilities (Wiener and Pazzaglia, 2021; Bubric et al., 2021). Drawing on ‘graphical psychology’, Li et al. (2024) revealed how design elements interact with the brain’s spatial processing system and emphasized environmental consistency, thereby optimizing spatial orientation behavior. They constructed a ‘Spatial Intervention Therapy Model’ from four aspects: spatial enclosure, wall design, furniture combination, and ground paving, providing concrete implementation schemes for environmental therapy. However, the interrelationship between spatial color, graphics, and individual spatial orientation abilities remains vague, stemming from medical scholars typically focusing on the cognitive impacts of visual information, while psychologists and designers from the architectural and spatial design fields generally study environment and behavior from the building and space design itself. Hence, few scholars have constructed relationships among color, imagery, and spatial orientation.

Recent examples have mainly focused on factors affecting individual spatial orientation abilities and concentrated on testing and comparing different cognitive tasks and indices (Muffato and De Beni, 2020; Coughlan et al., 2018; Fricke et al., 2021). These studies have revealed the close relationship between spatial orientation tasks and spatial configurations, raising two academic questions: (1) the interaction between spatial color and graphic elements and spatial orientation tasks; (2) how effective spatial interface designs can aid spatial orientation abilities. Although extensive experiments have provided evidence for the effects of spatial interventions on spatial orientation abilities (Li et al., 2024; Pouyan et al., 2021; Jamshidi et al., 2020; Delahaye et al., 2015), specific evidence for the potential and implementation pathways of spatial color and graphics remains lacking. Spatial attributes guide and constrain human behavior, yet they are not the sole determining factors. Studer (1970) argued that factors influencing individual behavior also include genetic characteristics, an aspect previously overlooked in studies.

The global public health event of COVID-19 has made exploring the goals of ‘ecological healthcare’ in sustainable architecture particularly important (Barbier and Burgess, 2020). Thanks to the site support provided by the Guangzhou Yuexiu Elderly Service Center and professional opinions from the Department of Neurology at the First People’s Hospital of Zhejiang City, this paper has a research basis in exploring ‘design methods of spatial color and graphics to aid patients in spatial orientation’. Research objectives: (1) To review through literature the potential and positive roles of color and graphic elements in spatial intervention aiding spatial orientation; (2) To investigate elderly patients’ preferred color spaces based on the CIE 1976 color system and using the analytic hierarchy process; (3) To propose design strategies to aid patient spatial orientation from the perspectives of text, graphics, and color through a case study. Hypotheses: (1) Layered and graded designs of spatial signage can help create spatial continuity and aid in navigation; (2) Distinct colors, simplified text, and graphic content can help patients recognize spaces. Due to limitations, the main constraints of this paper include the lack of behavioral experiments. Additionally, the selection of survey participants may also be insufficient. Besides visuospatial impairments, other complex factors affecting human spatial orientation abilities include visual factors. This paper seeks to strengthen cooperation with relevant medical institutions in future research and apply the proposed design concepts in practice. The proposed models of visual information and sensory stimulation stages in this paper will promote human-centered design in medical spaces and research into spatial cognition.

Research steps include: (1) To review the progress of research on spatial interventions for human spatial orientation ability to find the correlation between spatial orientation ability and environmental factors; (2) Extracting the overall color imagery of the second and fourth floors of the Yuexiu Elderly Service Center in Guangzhou based on the CIE 1976 color system and investigating color preferences of patients with mild cognitive impairment through the Analytic Hierarchy Process (AHP), to identify factors influencing their satisfaction with environmental colors; (3) Discussing the beneficial aspects of spatial color and graphic design in aiding patients’ orientation in space from public medical environments to home-style rehabilitation settings, with case analyses from macro to micro perspectives, and proposing effective design strategies; (4) Comparing these findings with relevant research to highlight the study’s value, exploring the implementation pathways, adaptability, and potential differences in practice, and finally suggesting improvements addressing the limitations.

## 2 A literature review related to spatial intervention in human spatial orientation ability

Contemporary scholars have focused on the impact of cognitive task training on human spatial orientation abilities, discussing this within the contexts of virtual and real-world environments.

In the realm of virtual environments, research has centered on developing technologically based training programs. Serino et al. (2017) proposed a novel virtual reality-based training scheme, designed to enhance the “mental frame synchronization” in Alzheimer’s patients, highlighting the potential of virtual reality in cognitive training for spatial orientation. Paruchuri et al. (2020) explored the potential of virtual training environments to enhance three-dimensional spatial cognition. Kober et al. (2013), utilizing a VR-based verbally-guided passive navigation training program, discovered significant improvements in spatial cognition in patients with spatial orientation disorders. Wollesen et al. (2020) linked virtual training to patients’ physical fitness, finding that physical training interventions, including dual-task elements, could address both egocentric and allocentric aspects of spatial orientation. Wadhwa and Walia (2018) observed the beneficial impact of Nintendo Wii Brain Training Games on the cognitive functions of young individuals, such as perceptual speed, working memory, and spatial orientation, though their effects on the elderly were not explored. Jansen et al. (2018), after administering weekly cognitive-motor coordination and core stability training to 28 office workers, noted improvements in mental rotation, spatial visualization, and cognitive processing speed, though the latter task’s effects were less clear. Interestingly, Paruchuri et al. (2020) found that two-dimensional training was as effective as three-dimensional training in enhancing spatial cognition.

In the dimension of physical environments, research has investigated the influence of environmental characteristics on spatial orientation. Levay et al. (2016) explored preferences for Euclidean rules and signal stimuli as spatial navigation strategies across different age groups and genders. Munoz-Montoya et al. (2019) emphasized the significant improvements that augmented reality can make in helping patients recall object locations and orient themselves spatially. Henderson et al. (2018) compared attentional spaces in two free-viewing scenes, discovering that cognitive relevance plays a dominant role in directing human attention in controlled scenarios. In studies of wandering behavior in spaces, the Architectural Institute of Japan found that cognitively impaired patients exhibited random wandering behaviors in empty spaces. However, their walking paths were influenced by spaces with prominent landmarks. Building on this, Weicong and Zhaoming (2020) further explored the impact of different spatial formations on the wandering behaviors of Alzheimer’s patients. Through spatial syntax experiments, they found that spatial layouts could guide such patients’ wandering behavior and discovered that centripetal layouts could lead patients to communal spaces to increase social interactions, thus stimulating brain activity and slowing cognitive decline.

In summary, research is abundant on the correlation between external physical environments and human spatial orientation abilities. However, studies on how to modify external physical environments to enhance spatial orientation capabilities are scarce, especially regarding specific implementation pathways and strategy models. Literature on the assistance of spatial color and graphic design in human spatial orientation is particularly limited, highlighting the research value of this paper.

## 3 Methodology

### 3.1 Pathological features of visuospatial disorders and the spatial orientation role of color and graphics

Visuospatial impairment is a common neurocognitive disorder characterized by compromised spatial orientation abilities (Costa et al., 2020; Derbie et al., 2022). This symptomatology tends to intensify with age, particularly prevalent among the elderly (Yin et al., 2015). Cognitive impairment progresses through mild, moderate, and severe stages, with worsening conditions correlating positively with declines in spatial orientation (Courtes et al., 2019), affecting patients’ ability to recognize directions, judge locations, and perceive spatial distances. The effectiveness of spatial interventions significantly diminishes at moderate and severe stages (Marquardt et al., 2014). Consequently, such interventions are deemed suitable for patients with Mild Cognitive Impairment (MCI), who represent the primary study population for this paper. These patients exhibit mild cognitive decline, which, while not severely impairing daily functions, still allows for independent living (Petersen et al., 1999). Early spatial interventions can significantly delay disease progression and enhance quality of life, thereby preventing or postponing the onset of severe cognitive impairments.

Patients in this category often exhibit difficulty in spatial orientation (Wang and Cao, 2017), inability to distinguish directions, and in locating objects. The patient also has some spatial neglect and three-dimensional visual–spatial deficits (Zhou and Cao, 2014), which make it difficult for them to judge where objects are in space (He et al., 2015). For instance, they may reach for objects too far, too quickly, or insufficiently, which could result in them falling over or missing their target. Additionally, this group of patients will also have macropsia or micro sightedness (He, 2003), which includes challenges determining the relationship between the volume and distance of two similar objects, visual confusion between three-dimensional and two-dimensional planes, and the inability to tell the difference between the depth relationship between the brightness proximity of light and dark surfaces.

The visual impact and emotional characteristics inherent in color graphics play a significant role in spatial orientation. Color is considered the most crucial element in spatial information guidance, accounting for 80% of initial visual responses (Sun et al., 2007), followed by graphics. The emotional characteristics of color graphics arise from sensory associations between the object and the individual, with people instinctively assigning symbolic meanings to various colors based on their personal “experience” within a comprehensive environment. Associating different colors with different spaces and objects can foster emotional resonance between individuals and corresponding spaces. According to the “7-s rule” proposed by the American Popular Color Research Center (Ware, 2019), a scientifically designed color system in hospitals can create a lasting visual impression, aiding medical staff and patients in navigating spatial information. Finland’s Hatanpaa Community Hospital uses colored floor markings to guide first-time visitors to specific areas (Chong et al., 2023). Current hospital signage systems categorize functional areas based on their importance and foot traffic, employing color coding for differentiation (Wang et al., 2002).

### 3.2 Analytical hierarchy process and research process

The Analytic Hierarchy Process (AHP), established by Saaty (1980) in the 1970s, is a systematic and hierarchical analysis method. It constructs multi-level structural models to unearth the relative importance of decision-making elements, offering a novel approach that combines qualitative and quantitative analysis for multi-criteria decision-making. This method has seen extensive application in color perception analysis (Huang and Guo, 2023). Compared to traditional intuitive judgment or single-variable analysis methods, AHP excels in assessing the relative importance of different color attributes in complex color systems, striking a balance between quantitative data and expert opinions.

Considering that factors influencing human behavior encompass both environmental and non-environmental elements, adopting a multidisciplinary research approach is more rational (Zeisel, 2006). Humanized warm color tones are widely applicable and conform to visual color aesthetics. Numerous studies indicate that older adults prefer a warm color environment, contrasting with the unsatisfactory cold color environments dominated by white and blue-grey in most hospitals (Peng, 2018). Thus, this study commenced with a user color preference survey at the Yuexiu Senior Service Center in Guangzhou using AHP, followed by a case study analysis of space interface design strategies beneficial for spatial orientation.

Research process: (1) utilizing the YS4560 spectrophotometer to collect CIE 1976 L*a*b* values from the second and fourth floors (main activity levels) of the Yuexiu Senior Service Center, and converting them into RGB color samples using relevant formulas; (2) conducting a spatial color perception survey among participants (dementia patients, caregivers, family members) using the Likert scale and verbal analysis method (Qiang and Fei, 2017). Dominant and semantic factors were extracted using semantic differential methodology, and evaluation criteria were established; (3) implementing weight analysis, consistency testing, and perception evaluation based on the AHP structural model to determine participants’ color preferences and to highlight prevalent environmental design issues in care institutions; (4) combining user color preferences to analyze space interface design from macro to micro perspectives in both public medical and home-care environments, proposing design strategies.

Contrary to some studies, this paper first measures colors in the CIE 1976 system and then converts them to RGB values before conducting color perception surveys, a process that is more scientifically sound. This study considers the visual perception characteristics and environmental factors of colors when extracting spatial color samples, instead of directly using RGB colors as perception samples. Due to the limitations of the RGB color system in brightness and saturation, and its display variability across different devices, it struggles to fully reflect the overall spatial color perception of individuals in terms of accuracy and consistency.

### 3.3 CIE 1976 color systems and measurement instruments

The Commission Internationale de l’Éclairage (CIE) established a globally recognized set of color standards to ensure consistency and accuracy in color representation (Jost et al., 2018). The CIE 1931 standard colorimetric system is considered foundational in colorimetry, delineating colors through three coordinates representing the perceived brightness and color distribution in human vision (Jiang and Mao, 2002). The CIE 1976 L*a*b* uniform color space, built on the CIE 1931 X , Y , Z system, employs nonlinear adjustments to the color space, making color differences more visually uniform and intuitive. For color analysis, the CIE 1976 system is particularly meaningful as it scientifically reflects the human eye’s observation of patterns such as camouflage, also offering greater universality. Measuring the overall color impression of a space using this system requires the use of relevant measuring tools for point measurements at different indoor locations to capture the L*a*b* values of surface materials. During the selection of measurement points, the significance of different areas or the calculation of mean values should be considered to represent the overall color impression of the interior.

Compared to RGB color systems, this paper selects the CIE 1976 color system for the elderly’s spatial color perception for the following reasons (Schwarz et al., 1987; Mehta, 2005; Nalini and Malleswari, 2016; Ngau et al., 2009): (1) In terms of color accuracy, the CIE 1976 system closely aligns with the nonlinear response of human vision, offering more detailed color interval differentiation that more accurately reflects visual color differences; (2) Regarding color consistency, the CIE 1976 color space is designed as a uniform color space, ensuring that the same color differences are perceived consistently within this space, aiding in maintaining objective and consistent color assessments in research; (3) For data analysis, the uniform nature of the CIE 1976 system makes color data more suitable for statistical analysis and pattern recognition, simplifying the color data processing workflow; (4) In cross-media applications, as the CIE 1976 color space is extensively utilized in color management systems, it guarantees color consistency across different devices and materials, crucial for studies involving diverse visual materials in the elderly’s color perception.

**Figure 1:**
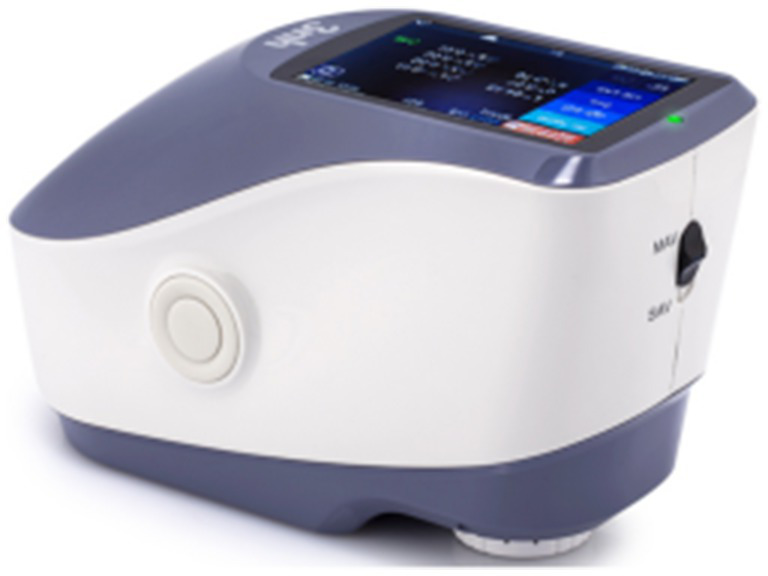
The YS4560 spectrophotometer.

Table 1 outlines the relevant specifications of this device (Figure 1), including its measurement accuracy, wavelength range, and measurement duration. The YS4560 spectrophotometer is a device specifically designed for high-precision color analysis, suitable for chromatic measurement of various materials, including textiles, plastics, and paints. It accurately measures the L*a*b* values of samples, providing key color data for evaluating and controlling color quality.

**Table 1:** 3nh San En Shi brand color measuring instrument parameters.

Model No. YS4560	Caliber: Φ48mm	Wavelength range and interval: 400 ~ 700 nm/10 nm
Half bandwidth: 10 nm	Measuring time: 1.5 s	Reflectance measuring range: 0 ~ 200%
Lighting Method: 45/0 (45 ring uniform illumination 0° reception)		Measuring way: single measurements, averaged (2–99)
Spectrometric Method: Concave grating spectral		Sensor: 256 pixel dual-array CMOS image sensor
Storage capacity: 500 Standard Samples, 20,000 Test Samples		
Operating temperature range: 0 ~ 40°C, 0-85%RH (no condensation), Altitude: below 2000 m		
Storage temperature range: −20-50°C, 0-85%RH (no condensation)		
Repeatability: Spectral reflectance: MAV, standard deviation within 0.1% (within 0.2% for 400-700 nm); Chromaticity values: MAV, △E*ab within 0.05 (after instrument warm-up and calibration, measured on a white plate 30 times at 5-s intervals for average value).		
Stand: Lighting method in accordance with the standard CIE No.15, GB/T 3978, GB 2893, GB/T 18833, ISO7724-1, ASTM E1164, DIN5033 Teil7		

## 4 Results

### 4.1 A survey on users’ color preference perception based on analytical hierarchy process

#### 4.1.1 User spatial color perception extraction

**Figure 2:**
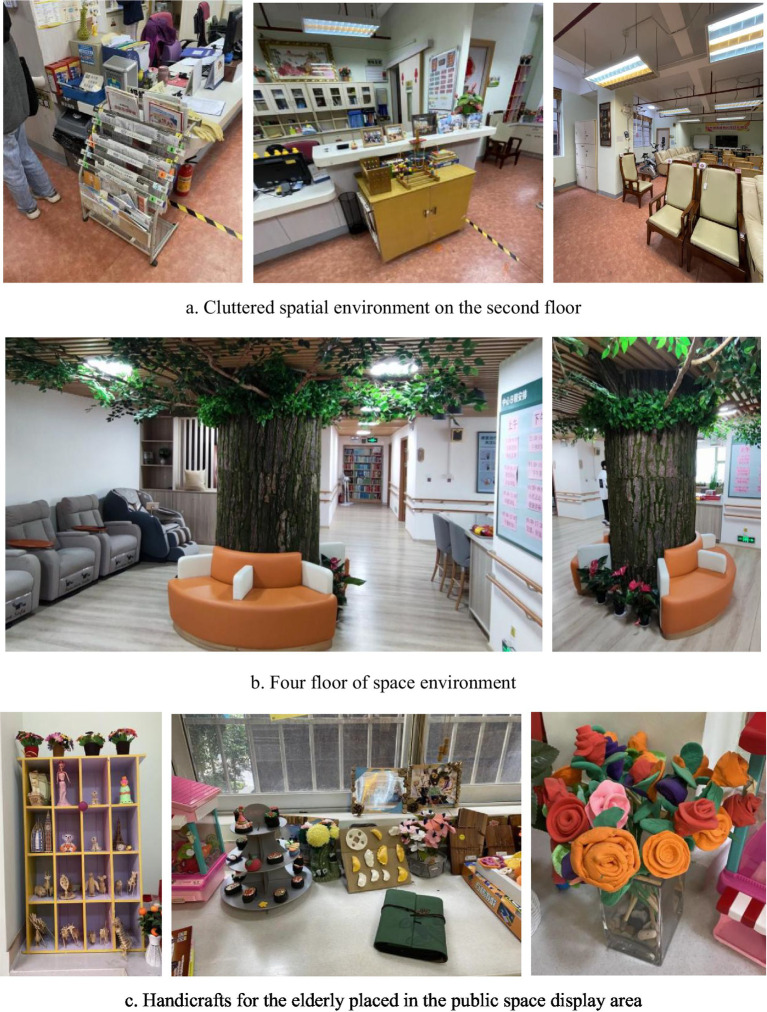
Spatial environment of the second and fourth floors of the service center. (a) Cluttered spatial environment on the second floor. (b) Four floor of space environment. (c) Handicrafts for the elderly placed in the public space display area.

The first step is extracting spatial color perception samples based on the CIE 1976 color system. Given the color intermixing in the second and fourth-floor spaces of the service center (Figure 2), it was more reasonable to extract colors occupying a larger area to represent the overall space. Additionally, for scientific rigor, the impact of similar colors on user perception of space was considered. Therefore, this paper measured the L*a*b* values of material interfaces occupying significant areas in the space, grouping them, and then converting the average values of each group into RGB color values. Three representative color groups were selected for measurement in the second-floor space, while four were chosen in the fourth-floor space. Following relevant requirements and measurement standards, the L*a*b* values and averages for each group were obtained (Table 2).

**Table 2:** Elemental grouping, fixed-point measurement and operation specification.

Grouping and measurement positions	Average value		
	L*	a*	b*
Level 2 space			
Group 1: Floor	59.73	23.93	22.28
Group 2: Lockers, some advertising wall, wooden tables	83.13	0.60	9.68
Group 3: White tables, walls, ceilings, fabrics	91.47	−1.04	1.81
Level 4 space			
Group 1: Ceiling wood grille	71.55	0.88	10.65
Group 2: Partial furniture & background wall	80.89	0.42	24.62
Group 3: Wooden floors, grills, fabric sofas	77.94	1.25	−1.27
Group 4: Plants	35.94	−28.59	34.71

Instrument Measurement Operation Method: (1) Instrument Warm-up and Calibration: Before conducting measurements, the instrument should be adequately warmed up to ensure its internal components reach a stable working state. Subsequently, perform standard calibration using a standard white plate or a standard sample with a known reflectance to ensure the accuracy of measurement data; (2) Sample Preparation: Ensure that the surface of the sample to be tested is clean and free from contamination, to prevent interference in measurement results caused by surface impurities or improper handling. The sample should be placed flat on a stable stand, ensuring that the test area is uniform and consistent; (3) Parameter Setting and Preliminary Testing: Set appropriate measurement parameters in the instrument according to the type of sample and its color characteristics to be measured, such as wavelength range, light source type, and observation conditions, etc. Conduct preliminary testing to ensure the normal operation of the equipment; (4) Measurement and Data Recording: Proceed with the measurement and record the data systematically.

Subsequently, L*a*b* values were converted into RGB color values using relevant formulas to extract effective, representative color samples. First, Equation 1 was used to convert the average L*a*b* values of each group into X , Y , Z values, where $X_{n}$ , $Y_{n}$ , $Z_{n}$ are the reference white point values for the standard D65 light source, respectively 95.047, 100.000, 108.883. Next, X , Y , Z values underwent linear transformation through Equation 2, where $R^{,}$ , $G^{,}$ , $B^{,}$ are the linear RGB values. This was followed by the inverse Gamma correction executed through Equation 3, resulting in corrected RGB values. Finally, these values were converted to the 0–255 range using Equation 4, yielding effective RGB color samples (Table 3).


(1) $$\begin{array}{c} \begin{array}{l} Y=Y_{n}\times {\left(\frac{L*+16}{116}\right)}^{3}\;\mathrm{for}\;L*>7.9996\;\mathrm{or}\;\\ \;\;\;\;\;\;\;\;\;Y=\frac{L*}{903.3}\;\mathrm{for}\;L*\leq 7.9996 \end{array}\\ X=X_{n}\times {\left(a*/500+\frac{L*+16}{116}\right)}^{3}\\ Z=Z_{n}\times {\left(\frac{L*+16}{116}-b*/200\right)}^{3} \end{array}$$

**Table 3:** Valid color samples from the second and fourth floors of Guangzhou Yuexiu Senior Service Center.

The second floor				The fourth floor			
Groups	RGB values	CIE 1976	Colors	Colors	RGB values	CIE 1976	Groups
1	R = 195; G = 127; B = 106	L* = 59.73 a* = 23.93 b* = 22.28			R = 188 G = 174 B = 156	L* = 71.55 a* = 0.88 b* = 10.65	1
2	R = 216; G = 206; B = 184	L* = 83.13 a* = 0.60 b* = 9.68			R = 219 G = 199 B = 155	L* = 80.89 a* = 0.42 b* = 24.62	2
3	R = 245; G = 231; B = 213	L* = 91.47 a* = −1.04 b* = 1.81			R = 215 G = 192 B = 183	L* = 77.94 a* = 1.25 b* = −1.27	3
					R = 52 G = 95 B = 23	L* = 35.94 a* = −28.59 b* = −1.27	4

In the equation, L∗ represents lightness, with a numerical range from 0 to 100; a∗ denotes the red-green axis of color, where positive values indicate a red bias and negative values a green bias; b∗ represents the yellow-blue axis, where positive values suggest a yellow bias and negative values a blue bias; X , Y , Z signifies the three components of the CIE 1976 color space; $X_{n}$ , $Y_{n}$ , $Z_{n}$ refers to the reference white point values of the light source.


(2) $$\begin{array}{c} R\prime =X\times 3.2406-Y\times 1.5372-Z\times 0.4986\\ G\prime =-X\times 0.9689+Y\times 1.8758+Z\times 0.0415\\ B\prime =X\times 0.0557-Y\times 0.2040+Z\times 1.0570 \end{array}$$

In the equation, X , Y , Z designates the three components of the CIE 1976 color space; $R^{\prime }$ , $G^{\prime }$ , $B^{\prime }$ represents the values linearly transformed from RGB, with a range from 0 to 1.

(3) $$\begin{array}{l} R=12.92\times R\prime \mathrm{for R}\prime \leq 0.0031308\mathrm{or R}=1.055\times R\prime^{1/2.4}\\ \;\;\;\;\;\;\;\;\;\;\;\;\;\;\;\;\;\;\;\;\;\;\;\;\;\;\;\;\;\;\;\;\;\;\;\;\;\;\;\;\;\;\;\;\;\;-0.055\;\mathrm{for R}\prime >0.0031308\\ G=12.92\times G\prime \mathrm{for G}\prime \leq 0.0031308\mathrm{or G}=1.055\times G\prime^{1/2.4}\\ \;\;\;\;\;\;\;\;\;\;\;\;\;\;\;\;\;\;\;\;\;\;\;\;\;\;\;\;\;\;\;\;\;\;\;\;\;\;\;\;\;\;\;\;\;\;\;-0.055\;\mathrm{for G}\prime >0.0031308\\ B=12.92\times B\prime \mathrm{for B}\prime \leq 0.0031308\mathrm{or B}=1.055\times B\prime^{1/2.4}\\ \;\;\;\;\;\;\;\;\;\;\;\;\;\;\;\;\;\;\;\;\;\;\;\;\;\;\;\;\;\;\;\;\;\;\;\;\;\;\;\;\;\;\;\;\;\;\;-0.055\;\mathrm{for B}\prime >0.0031308 \end{array}$$

In the equation, R , G , B is the standardized value transformed from linear $R^{\prime }$ , $G^{\prime }$ , $B^{\prime }$ into the RGB color system.


(4) $$R_{255}=R\times 255,G_{255}=G\times 255,B_{255}=B\times 255$$

In the equation, $R_{255}$ , $G_{255}$ , $B_{255}$ refers to the RGB values commonly used in digital displays and cultural image media, which range from 0 to 255.

#### 4.1.2 User interviews and extraction of spatial color perception factors

The second step is interviewing users and using oral analysis (Qiang and Fei, 2017) method to extract spatial color perception factors. The method is a qualitative research technique that gathers participants’ subjective cognition and feelings through verbal feedback. This method allows for the direct capture of users’ initial reactions and emotional associations with environmental colors during experiments, thereby enabling researchers to more accurately identify key factors in color perception. In the selection of the participant group, this study conducted preliminary color cognition tests using image displays to ensure that participants could engage effectively in subsequent color perception experiments. Out of the 27 patient participants, three were excluded due to apparent deficiencies in color recognition. Although the remaining 24 participants exhibited varying degrees of visual–spatial impairment, it was challenging for the team to fully investigate the impact of other ocular diseases on color perception. As mentioned previously, the factors influencing elderly patients’ perception of spatial colors are complex, potentially including color weaknesses, hyperopia, myopia, or other eye diseases. Given the inclusivity considerations of the experimental design, the study did not pursue further in-depth investigation of these 24 participants, as this would not impede the progress of subsequent experimental designs. On the contrary, the study posited that the color perception experiment results of these patients would be more inclusive and reflective of the broader population’s true circumstances.

**Figure 3:**
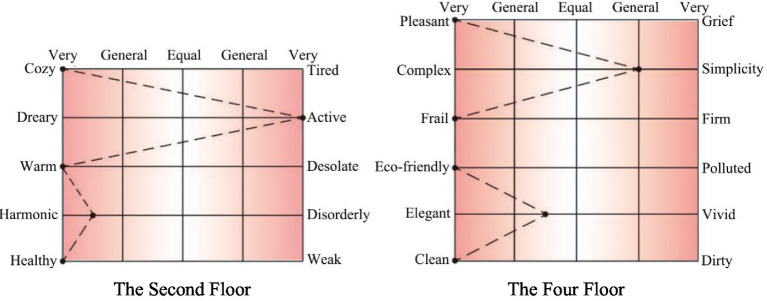
Description of the color environment intentional vocabulary on the second and fourth floors of Guangzhou Yuexiu Senior service center.

This study selected the period of 2:30–4:30 PM (the peak of the activity, and the communication selection target is wider) to communicate with 24 patients and 6 caregivers in a guided manner, and extracted a total of 86 effective perceptual imagery words (42 in the second level and 44 in the third level) from the interviewees’ oral narrators. Then, the valid vocabulary was categorized into 24 groups of representative semantic elements (10 groups in the second level and 14 groups in the fourth level), and after inviting 12 design-related professionals (6 university teachers, 2 professors, and 4 people in the industry) to make scores, the 24 groups of vocabulary were downscaled by principal component analysis to screen the groups of vocabulary with too similar semantics, and the antonymic repertoire groups were constructed simultaneously. Finally, the semantic difference method (Osgood et al., 1957) was used to quantitatively analyze the semantic repertoires, construct a five-point psychological scale, and invite the subjects to perceptually evaluate the extracted spatial color samples, with the evaluation indexes divided into five semantic level changes of −2, −1, 0, 1, and 2, corresponding to very, average, equal, average, and very. After the subjects completed the evaluation, the characteristic semantic description map was obtained using a line graph (Figure 3).

Regarding the medium for color perception, this paper employed Apple brand iPads for display. If printed paper media were used, color samples could easily become distorted, directly affecting users’ color perception. The advantages of using electronic screen displays include (1) providing higher color resolution and a wider color gamut range, ensuring vividness and richness in colors; (2) the adaptive adjustment capability of the elderly’s ocular nerves to electronic screen light sources is relatively low, while electronic screens can be adjusted to a color brightness more comfortable for participants; (3) the portability and ease of use of electronic screens also make it possible to conduct color perception research in various environments and contexts, further expanding the research’s application scope and practical value.

According to the measured survey data, a total of 11 effective environmental color imagery words were obtained, which are cozy, dreary, warm, harmonic, healthy, pleasant, complex, frail, eco-friendly, elegant, and clean. One of the terms representing the second tier is warm, cozy, harmonic, and healthy. The words that represent the fourth level are eco-friendly, elegant, and clean. The common words representing the second and fourth levels are cozy, active, harmonic, warm, and healthy.

#### 4.1.3 Color weighting analysis and perceptual evaluation based on analytical hierarchy process

**Figure 4:**
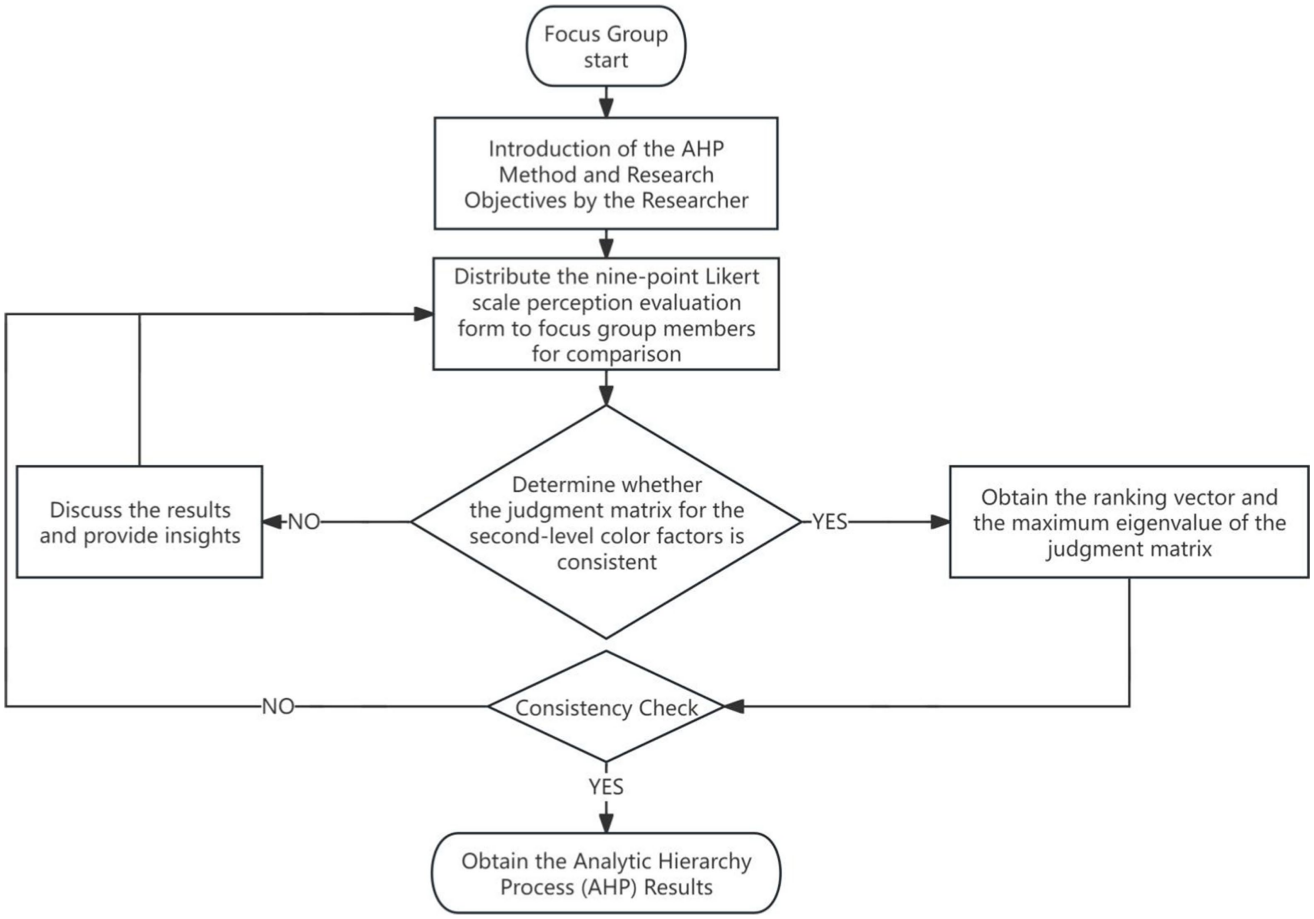
Focus group discussion flowchart.

The third step involves the analysis of color weight and perceptual evaluation. The AHP is applied to encode and analyze (Wu and Yang, 2019) the weight of color samples and their associated affective lexicon listed in Table 3, constructing two-tier and four-tier maps of dominant and semantic factors (Tables 4, 5). Three experts from the field of design and relevant stakeholders (one service center manager and two elderly individuals) were invited to participate in a focus group discussion. The purpose of this discussion is to assign weights to the extracted color factors in order to rank their importance, thereby identifying the color environment characteristics preferred by elderly individuals. Figure 4 illustrates the detailed process of the focus group discussion (Davis et al., 2018). Initially, the researchers introduced the AHP method and the study’s objective to the group members, and distributed the nine-point scale perceptual evaluation form (Table 6) to the participants. After all members completed the survey, the researchers used their feedback to derive the factor judgment matrices for the second and fourth floor spaces (Table 7). Following this, the group members were asked to conduct pairwise comparisons of the relative importance of each factor using the nine-point scale method. This process was conducted in two rounds: in the first round, each member independently assessed the color dominant factor judgment matrices for the two floor spaces. In the second round, the group members discussed the items with significant discrepancies identified in the first round, exchanged views, and reached a consensus to reconstruct the judgment matrices. Finally, the weights of the factors were calculated and a consistency test was conducted to ensure the scientific rigor and reliability of the results.

**Table 4:** Mapping of explicit and semantic factors of the second color environment.

Codes	Effective color samples	CIE 1976	Codes	Environmental color imagery vocabulary
A1		L* = 71.55; a* = 0.88; b* = 10.65	B1	Warm
A2		L* = 91.47; a* = −1.04; b* = 1.81	B2	Cozy
A3		L* = 59.73; a* = 23.93; b* = 22.28	B3	Harmonic
			B5	Active

**Table 5:** Mapping of explicit and semantic factors of the fourth color environment.

Codes	Effective color samples	CIE 1976	Codes	Environmental color imagery vocabulary
A4		L* = 80.89; a* = 0.42; b* = 24.62	B6	Simplicity
A5		L* = 71.55; a* = 0.88; b* = 10.65	B7	Pleasant
A6		L* = 77.94; a* = 1.25; b* = −1.27	B8	Frail
A7		L* = 35.94; a* = −28.59; b* = −1.27	B9	Clean
			B10	Elegant
			B11	Eco-friendly

**Table 6:** Meaning of the number 1 to 9 scale in hierarchical analysis.

Scales	Meaning
*a_ij_* = 1	Element *i* has the same importance as element *j* for the previous level of factors
*a_ij_* = 3	Element *i* is slightly more important than element *j*
*a_ij_* = 5	Element *i* is more important than element *j*
*a_ij_* = 7	Element *i* is much more important than element *j*
*a_ij_* = 9	Element *i* is extremely more important than element *j*
*a_ij_* = 2,4,6,8	The importance of elements *i* and *j* is somewhere in the middle of adjacent judgments
*a_ij_* = 1/*a_ij_*	If the ratio of the importance of factor *j* to factor *i* is *a_ij_*, then the ratio of the importance of factor *i* to factor *j* is *a_ij_* = 1/*a_ij_*

**Table 7:** Judgment matrix for pairwise comparison of color dominant factors and hierarchical analysis results.

The second floor				The fourth floor				
Factors	A1	A2	A3	Factors	A4	A5	A6	A7
Judgment matrix								
A1	1	2	4	A4	1	2	3	4
A2	1/2	1	2	A5	1/2	1	2	3
A3	1/4	1/2	1	A6	1/3	1/2	1	2
				A7	1/4	1/3	1/2	1

The second floor				The fourth floor			
Factors	*W*	*CI*	*CR*	Factors	*W*	*CI*	*CR*
Hierarchical analysis results							
A1	0.57	0.005	0.01	A4	0.466	0.0002	0.00018
A2	0.29			A5	0.277		
A3	0.14			A6	0.161		
				A7	0.099		

The focus group discussion was open-ended, primarily aimed at assigning weight to the extracted color perception factors. However, before the discussion, the moderator initiated a warm-up session (Hennink, 2013) to enhance participants’ understanding of the color factors and to improve the accuracy of color weighting. The key questions included: (1) What are the overall color impressions and characteristics of the two floors? Are there perceptual differences in the color preferences between caregivers, elderly individuals, and family members? (2) Which color perception factor samples are preferred by the elderly across the two floors? (3) Do spatial decoration elements such as family portraits, personalized photographs, or memorabilia help stimulate cognitive recognition and balance the emotional state of the elderly? (4) What is the impact of color perception on the comfort and cognitive load of elderly individuals, particularly concerning the practical application of high brightness and low saturation colors? (5) How can the design holistically consider both visual aesthetics and functional needs, ensuring that color not only enhances spatial appeal but also caters to the specific cognitive requirements and emotional preferences of the elderly population?

In the judgment matrix constructed for the color samples, designated as $‘A=(a_{ij})n\;\cdot \;n’‚$ the values for $‘a_{ij}>0;i=1,2,\ldots ,n;a_{ij}’$ are determined using a nine-point scale method for perceptual evaluation, as detailed in Table 6. This scale ranges from 1 to 9 and includes their reciprocals. Higher scale values indicate that the factor *i* is considered more important than factor *j*. Thus, in environmental color design, greater consideration should be given to factor *i*.

Subsequently, focus group members were instructed to conduct pairwise comparisons of factors’ importance using a nine-point scale method of perceptual evaluation. This process was divided into two rounds. In the first round, each member was required to construct judgment matrices for both the second and fourth tiers of color saliency factors. Researchers collected 14 matrices and conducted statistical analyses on-site. In the second round, focus group discussions were held on options with significant disparities. Each member expressed their views on these options, and consensus was ultimately reached. The agreed-upon judgment matrices were then used to calculate weights.

Firstly, the above constructed judgment matrix and the sorting vector W of each judgment matrix were combined with Equation 5 to calculate the approximate solution $W={\left(W_{1},W_{2},W3,\ldots ,Wn,\right)}^{T}$ of the feature vector. Then, the maximum eigenvalue $\lambda_{\max}$ was calculated according to Equation 6, and the consistency index value CI was calculated by Equation 7 to test and correct the judgment matrix, and n in equation is the dimensionality of the judgment matrix. Finally, the random consistency ratio value CR is calculated by Equation 8, where RI is the average random consistency index of the judgment matrix and n is the order of the judgment matrix.

The Ranking vector W can be obtained by Equation 5.


(5) $$W_{i}=\frac{\underline{w_{i}}}{\sum_{i=1}^{n}\underline{w_{i}}}\left(i,j=1,2\ldots n\right)$$

In the given equation, $W_{i}$ represents the normalized weight vector W , which indicates the relative importance of a factor within the judgment matrix; $w_{i}$ denotes the sum of the elements in the original judgment matrix; n refers to the size of the judgment matrix, which is the total number of elements in the matrix.

The maximum eigenvalue $\lambda_{\max}$ can be obtained by Equation 6.


(6) $$\lambda_{\max}=\frac{1}{n}\sum_{i=1}^{n}\frac{{\left(CW\right)}_{i}}{W_{i}}$$

In the given equation, $\lambda_{\max}$ represents the maximum eigenvalue, which is used as an indicator to assess the consistency of the judgment matrix; CW stands for the weighted sum vector, which results from the multiplication of the original judgment matrix by the weight vector W ; n denotes the size of the judgment matrix, that is, the total number of elements within the matrix.

The consistency index value CI can be obtained by Equation 7.


(7) $$CI=\frac{\lambda_{\max}-n}{n-1}\;$$

In the given equation, $\lambda_{\max}$ represents the maximum eigenvalue, which is derived from the eigenvalues of the judgment matrix and reflects the overall consistency of the matrix; n denotes the size of the judgment matrix, that is, the total number of elements within the matrix; CI is an indicator used to measure the consistency of the judgment matrix. The smaller this value, the better the consistency of the matrix.

The random consistency ratio value CR can be obtained by **Equation 8**.


(8) $$CR=\frac{CI}{RI}$$

In the given equation, CI represents the index used to measure the consistency of the judgment matrix; RI is the average consistency value of a random matrix based on its dimension n . This value is utilized to normalize CI , enabling comparison across matrices of different sizes; CR is the criterion used to assess whether the consistency of the judgment matrix is acceptable. If this value is less than or equal to 0.1, the consistency of the matrix is considered acceptable.

The two-level and four-level judgment matrices of color dominant factor were constructed by nine-level scaling method (Table 7), and the weight vectors W , CI , CR of each judgment matrix were calculated to obtain the results of hierarchical analysis of color dominant factor. From the analysis results, all the CI and CR values are less than 0.1, thus proving that the ranking results of this hierarchical analysis method have satisfactory consistency. Among the seven valid color samples, the color samples with higher brightness have higher importance.

In the second round, the semantic factors were subjected to hierarchical analysis following the described steps, resulting in a judgment matrix and the outcomes of the analysis (Table 8). The results indicated that both the CI and the CR were below 0.1, confirming that the ranking results of this hierarchical analysis possess satisfactory consistency. In the analysis of the second-tier color semantic factors, “warm” was identified as the most representative; in the fourth-tier color semantic factors, “simplicity” emerged as the most representative.

**Table 8:** Results of the hierarchical analysis of the color semantic factor.

Factors	B1	B2		B3	B4	B5
Judgment matrix for pairwise comparison of common color semantic factors in the second and fourth layers						
B1	1	2		2	3	4
B2	1/2	1		2	3	4
B3	1/2	1/2		1	2	3
B4	1/3	1/3		1/2	1	2
B5	1/4	1/4		1/3	1/2	1
Pairwise comparison judgment matrix of color semantic factors in the fourth layers						
B6	1	3	5	2	4	7
B7	1/3	1	2	1/5	1/2	5
B8	1/5	1/2	1	1/7	1/5	1
B9	1/2	5	7	1	4	6
B10	1/4	2	5	1/4	1	3
B11	1/7	1/5	1	1/6	1/3	1

	*W*	*CI*	*CR*		*W*	*CI*	*CR*
Results of the hierarchical analysis of the color semantic factor.							
B1	0.36	0.002	0.002	B6	0.35	0.081	0.065
B2	0.28			B7	0.10		
B3	0.18			B8	0.05		
B4	0.11			B9	0.31		
B5	0.07			B10	0.15		
				B11	0.04		

### 4.2 Approaches to interface color graphics design that help people with visuospatial impairments orient themselves to space

#### 4.2.1 Color and graphic interface design for public healthcare environments case study

Based on previous studies that employed the hierarchical analysis method for environmental color characteristics, this paper will investigate the topic from three perspectives: public environments, home care environments, and the decorative elements of everyday items. The study explores the potential value of color patterns in providing spatial cues and assisting navigation tasks across various dimensions, such as corridors, walls, ceilings, floors, signage, and physical forms. It is important to note that the case study conducted in this section did not involve participant experiments.

**Figure 5:**
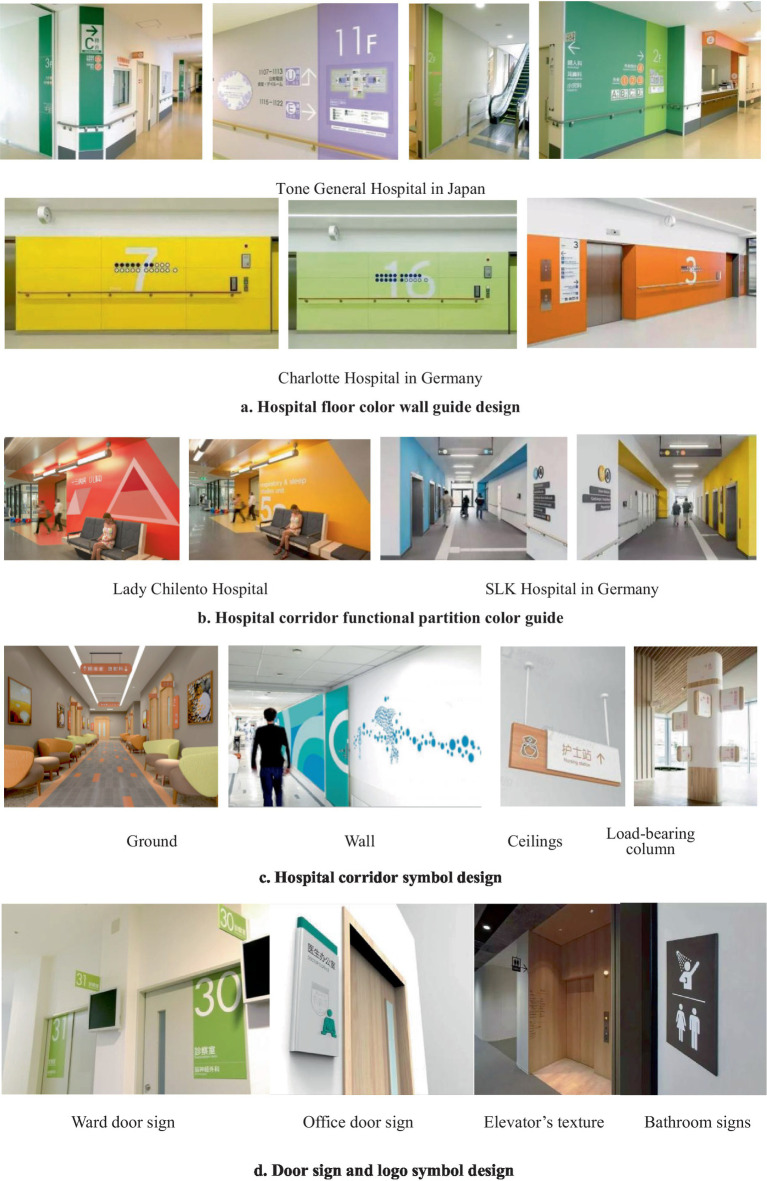
Spatial interface color and graphic and wayfinding design. (a) Hospital floor color wall guide design. (b) Hospital corridor functional partition color guide. (c) Hospital corridor symbol design. (d) Door sign and logo symbol design.

The impression effect of color association can link colors to spatial concepts, making it easier for visuals to swiftly discern spatial information and help people with weak spatial orientation find space (Ware, 2019). For instance, the Tone General Hospital in Japan (MHD MBC, 2023) and the Charlotte Hospital in Germany (Zhiren Wenbo, 2022) use color impressions to match each level, so even if you are not looking at the floor number, you can already have an idea of what floor it is (Figure 5). The Lady Cilento Hospital (RBLBCC, 2023) and the SLK Hospital in Germany (Zhiren Wenbo, 2022) are two further instances, both of which use local color to direct users to local functional partitions. Color images and symbols on the floor, walls, ceiling (Pre-Labeling, 2020), columns (SME CKGSB, 2017), room doors, and other spatial interfaces all play a role in providing further guidance as the patient approaches the target environment. The layout of the ward and office door numbers (Shenghemei Design, 2021), the color and texture of the elevator chambers, and the restroom signage (Zhiren Wenbo, 2022) will help patients locate their rooms more precisely and conveniently.

#### 4.2.2 A case study of interface design for a home-based rehabilitation environment

**Figure 6:**
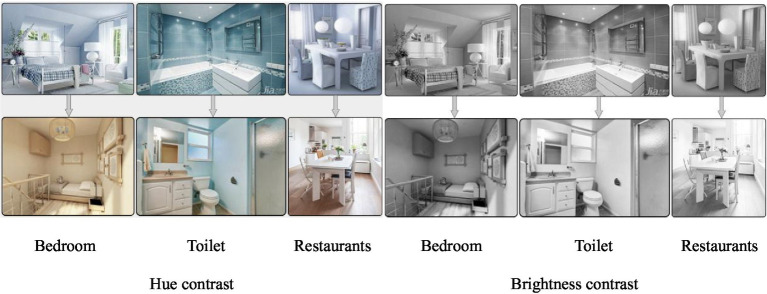
Color and brightness contrast analysis of the bedroom, bathroom and dining room.

The “family-style” care space can have a distinct overall color tone for the more often used spaces, such as the bedroom, dining room, and bathroom, but more thought should go into the hue and brightness. According to Figure 6, if the dining room, bathroom, and bedroom all have cold hues as their primary color, the patients may find it difficult to distinguish the rooms’ functions and locations.

On the other hand, the procedural memory created by being there for a long time lowered the likelihood of misorienting the area when each of the three spaces was given a hierarchical and soft color. In particular for areas with obvious topographical undulations (stairs, threshold stones), close spatial brightness was not conducive to patients’ ability to discriminate between spatial distance and depth. Additionally, patients with visual–spatial impairment may confuse two-dimensional planes and three-dimensional spaces, increasing the risk of tripping and falling. Contrarily, using distinct colors for the interface can help patients recognize ground barriers visually, improve their sense of spatial orientation, and lower their risk of falling.

However, the effect of color impressions may not apply to patients who also suffer from other eye diseases (e.g., glaucoma or red-green color blindness). Medication misuse as a result of this group of patients’ inability to discriminate between medications with similarly colored liquids or bottles of a similar shape and color is not unusual. Patients often discriminate between medications based on visual impression and memory, and even healthcare professionals may misidentify colorless liquid pharmaceuticals in the hospital, even though they are correctly placed. This is especially true when a doctor’s prescription is not written clearly. Between 1999 and 2017, the National Center for Injury Prevention and Control (2019) reported that more than 700,000 individuals in the United States died due to drug abuse annually. According to the latest data released by the U. S. Centers for Disease Control and Prevention (2024), over 1.5 million people in the U.S. visit the emergency department each year due to adverse drug events (ADE), with nearly 500,000 requiring hospitalization. Notably, more than 600,000 of these cases involve elderly patients aged 65 and above, more than twice the number of younger individuals.

Therefore, a single “visual memory impression” through color alone is limited, and the importance of graphic association is second only to the color element, which is composed of two parts, respectively, the external edge form and the internal message (Li et al., 2017). For example, the “+” sign of the red cross logo, even for color-blind people, will remind people of medical treatment, rescue, and other impressions. Classifying drugs according to the role of color + graphic association can control the possibility of drug misuse at the source.

This study argues that appropriate color and graphic design can assist patients in forming lasting memories of everyday items, particularly in the recognition of medication bottles and drugs. Table 9 illustrates the differences in color and shape among three groups of medication bottles and their visual impact on patients. In the shape group, Bottle D had more distinct shape characteristics compared to the other bottles, making it easier to recognize. In the color group, Bottle E, with its warm yellow color, stood out more compared to the cooler-colored bottles. After redesigning the bottles with appropriate use of color and shape, each group of bottles provided patients with a stronger visual memory. Therefore, the strategic use of shape and color in design can significantly aid patients in accurately identifying the functional properties of medications.

**Table 9:** Comparative analysis of the identification of the shape and color of the medicine bottle.

Process	Shape contrast	Color contrast	Mixed contrast
Groups	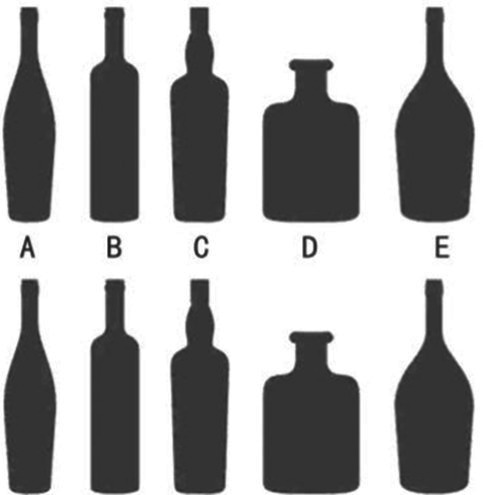	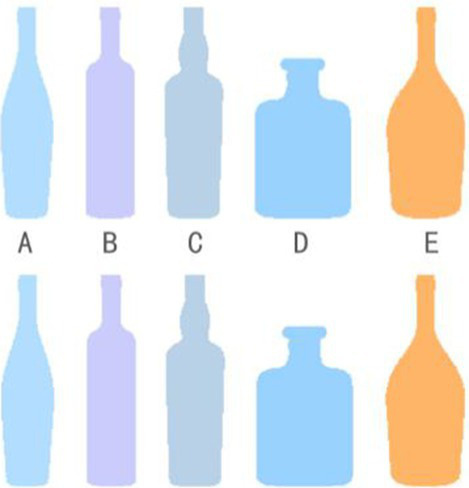	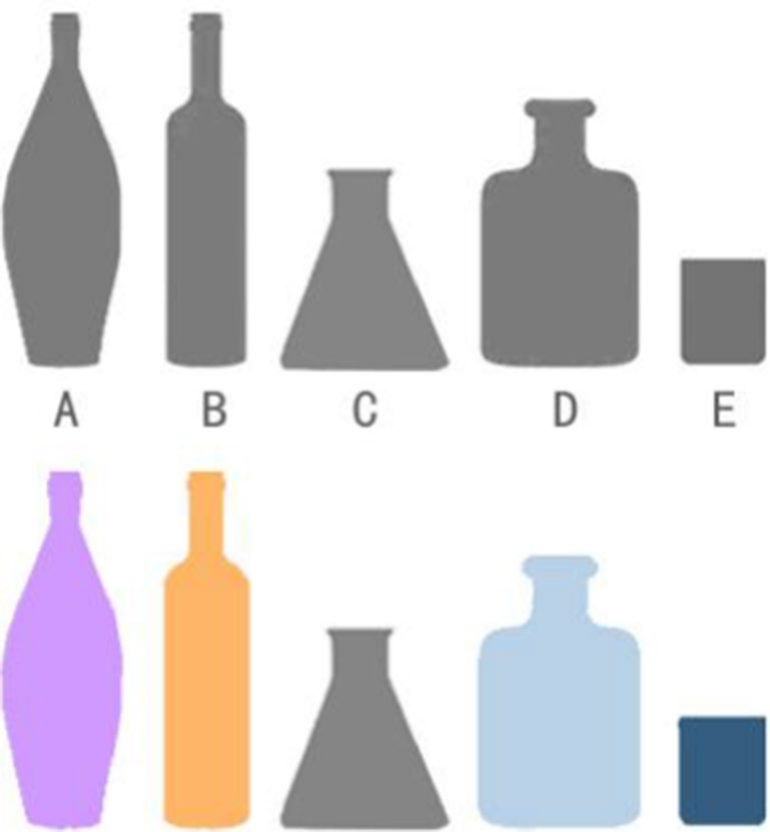
Recognize			
Features	A, B, C = Similar	A,C, D = Similar	All bottles have a high level of identifiability
	E = Confused	B = Confused	
	D = Highly recognizable	E = Highly recognizable	

Several studies offer empirical support for these conclusions. Based on focus group discussions and medication identification tests, Cardarelli et al. (2011) conducted an intervention study involving 25 elderly participants, all over the age of 65, recruited from over 130 clinics, to assess the effects of medication recognition. The study found that excessive labels and graphical information hindered patients’ ability to correctly identify medications. Although color coding proved effective, the selection of colors must avoid confusion with the color of the medication itself. Aceves-Gonzalez et al. (2024) used a modified version of the Human Error Assessment and Reduction Technique (HEART) to analyze a medication administration task in a simulated Neonatal Intensive Care Unit (NICU) scenario. This study demonstrated that a combination of color differences and personalized label design significantly reduced the risk of medication errors compared to traditional labels, particularly in emergency and critical care settings. PharmDs (2024) showcased innovations that improve medication recognition by altering the shape of medication bottles, using eco-friendly materials, and redesigning bottle caps. These improvements not only reduce the risk of medication misuse but also enhance patient compliance. Through constructing a deep learning-based model for blister-packaged drug identification, Ting et al. (2020) collected 250 types of blister-packaged drugs from the Out-Patient Department (OPD) of a medical center for comparative analysis. The study found that blister packaging with similar appearances increased the likelihood of medication errors, while the appropriate use of morphological features in drug packaging significantly reduced this risk.

## 5 Discussions

### 5.1 Discussion of results

The results of the hierarchical analysis showed that most seniors overall preferred brighter, simpler, clean, and warm colors, in line with the views of Serra et al. (2022), and Gashoot (2022). However, the following two findings in this study are noteworthy: (1) the design professionals who accompanied the on-site study thought that the fragmented decoration made the interior environment look cluttered (Figure 2), while some of the elderly felt that the environment was cozy; (2) the caregivers and design professionals generally thought that the decorative fake trees on the fourth floor lacked aesthetics, but some patients expressed a “satisfied “attitude. The survey results showed that patients’ satisfaction with the environment included not only the colorful environment visible to the naked eye, but also the artistic atmosphere, furnishing and decoration, and emotional memories (Harris et al., 2002). Most of the interior walls and display areas on the second and fourth floors display photos of the elderly’s handicrafts, outdoor activities, and family photos, and these intrinsic factors of emotional support occupy a non-negligible weight in the elderly’s perception of satisfaction with the environment.

For public healthcare environments, the design of spatial interfaces for beneficial patient identification functions and orientation spaces should be divided into four tiers: (1) primary signs are to give patients an overall grasp of the target orientation, such as setting different color tones for each floor, which helps patients distinguish the main functions of the space through color; (2) secondary signs are to use the signs on the ground, walls, ceilings, columns and other transitional spaces such as corridors to build a continuity of space guidance; (3) Tertiary identification is to accurately locate a small area of space through color or spatial pattern, such as using a bright uniform color at the front door of the nursing unit to enhance the visual impression, so that when patients recognize this color, they associate it with the ward area; (4) Tertiary identification is to find the specific target location through local identification after patients have accurately reached a small area. For example, adding signs on different ward doors, hanging small wooden signs (with the patient’s name), or attaching the patient’s favorite color to the room door can help patients identify their room quickly and accurately.

For home-based healing environments, functional areas should be given high brightness, low saturation, and colors with clear hue distinctions. In terms of household items, to strengthen the cognitive patient’s memory of the functional use. Similar substances should be differentiated by the differences in product shapes and colors so that patients can accurately identify the functional nature of the supplies.

Visual dominance seems to persist in human perceptual and cognitive systems, especially concerning visual information from spatial interfaces (Rucci et al., 2018). However, for patients with visual–spatial impairment, accurately acquiring spatial information through visual senses poses significant challenges. The sensory and cognitive processes begin with a momentary experience, followed by the presentation of details (Luo et al., 2022). The transition from color graphics to cognitive understanding involves a process of emotional conversion, influenced by personal experience, and vice versa for rational conversion. Therefore, designers should simplify the information at the forefront of the patient’s cognitive information process (guide interface information), including images, colors, textual information, and interactive interfaces.

**Figure 7:**
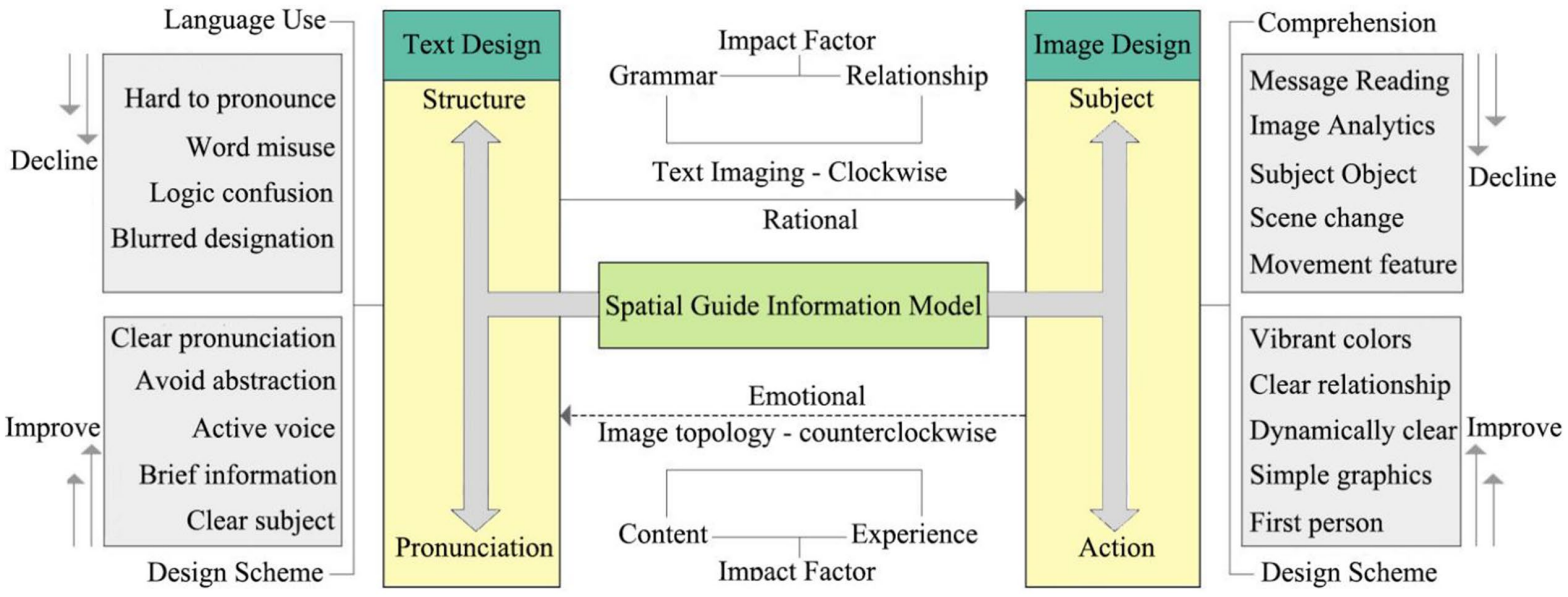
Spatial guide interface design model for patients with visual–spatial impairment.

Elderly people are cognitively more inclined to simple correspondence matching. Figure 7 shows the design model constructed in this paper for beneficial patients to recognize spatial visual information: (1) For image information, it should be concise and avoid using complex, abstract images to prevent the presence of multiple subjects and objects (Adesİna et al., 2022). (2) For human images, they should have obvious dynamic characteristics, and action features can be exaggerated to emphasize the intended message (Chai et al., 2019). The use of colors should be straightforward, avoiding multicolors, similar colors, dull colors, and highly saturated colors. (3) For graphic information, it should include clear directional indications, with color choices considering elderly patients with ocular diseases (e.g., low color sensitivity) (Ruzickova and Kroupova, 2020). (4) For textual information, avoid overly lengthy, complex, and obscure grammatical structures. Use concise declarative sentences, reduce unnecessary modifiers, and primarily use the active voice to create a direct correspondence between the subject and the object (Ignatova et al., 2023). (5) For the interactive interface, simplify the operation process, and ensure a clear and distinct figure-ground relationship in the interface’s graphics and text information (Kim et al., 2021).

**Figure 8:**
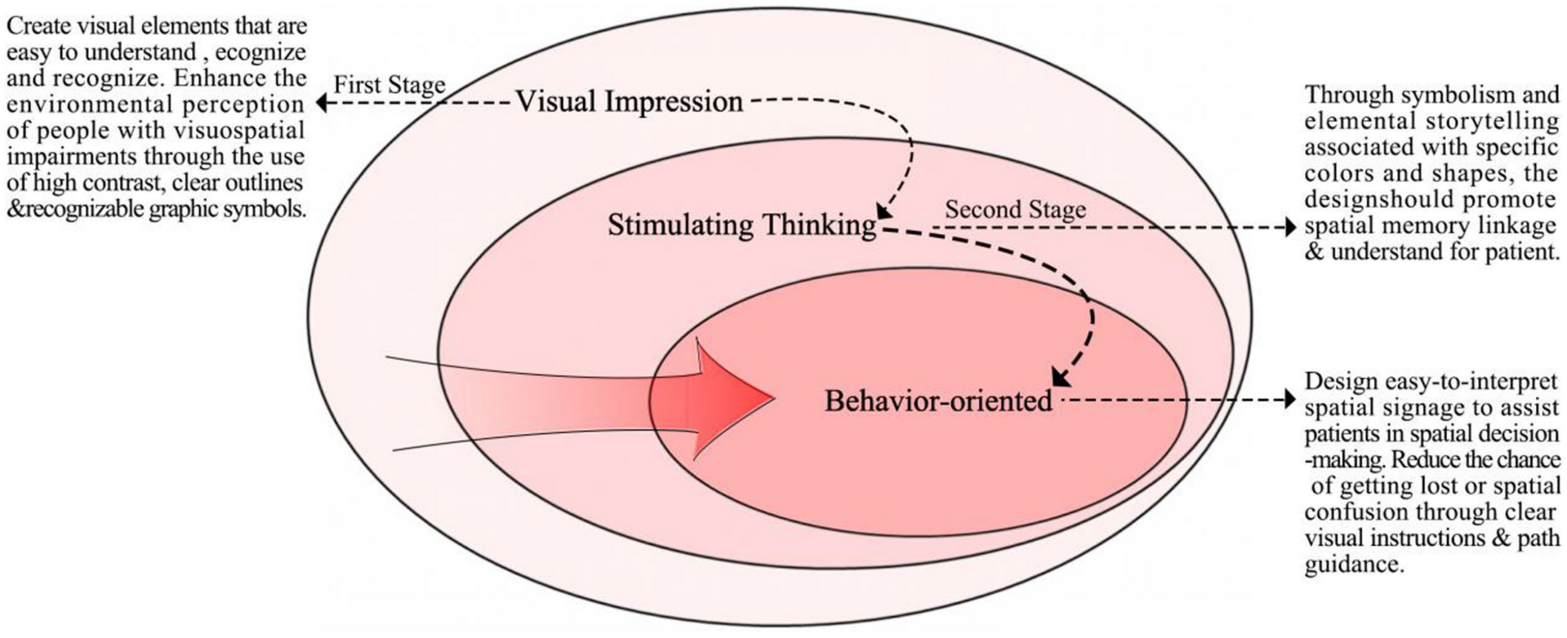
The realization stages of spatial color and graphic design assists patients in orienting to space.

Figure 8 displays the three stages of implementation proposed in this paper regarding interface color graphic design to assist spatial orientation for people with visuospatial impairments: (1) The first stage, “visual impression,” enhances the impact of spatial attributes on visual perception by amplifying the contrast and saturation of key visual elements to improve patient perception and recognition of spatial layouts and directional signs. (2) The second stage, “stimulating thinking,” leverages the emotional characteristics and metaphorical functions of color graphics to induce associative memory in patients, thereby creating a coordinate system for different spatial locations. For example, designing maps that include symbolic patterns representing the function or history of places while simplifying the layout to reduce cognitive load. (3) The third stage, “behavior-oriented,” strengthens environmental cues and integrates multisensory information to form an intuitive navigation system, thereby promoting spatial behavior in patients. For instance, guiding visually impaired individuals along safe paths through changes in tactile flooring or enhancing spatial orientation intuitiveness with auditory and lighting cues.

### 5.2 Research values

This article responds to two questions posed in the introduction: (1) The interplay of spatial color and graphic elements about spatial orientation tasks; (2) How effective spatial interface design can aid spatial orientation skills. The emotional characteristics of color graphics have a displacement and interactive relationship with human perception. Due to visual function deficits, patients with visual–spatial impairment have higher requirements and desires for visual stimuli. For patients with spatial orientation difficulties, visual guidance can be strengthened through the stimulation of color graphics, and narrative design can be used in the process of path guidance to activate brain neurons and effectively slow cognitive decline. This study’s results respond to the anticipated hypothesis and objectives: Spatial color graphics have the potential to improve spatial orientation abilities.

Based on the Lüscher Color Test, Stanzani Maserati et al. (2019) found that gray and black are rejected colors, while brown and gray are common choices for dementia patients. This result aligns with the findings of this article, although the former discovered that MCI patients are more anxious when facing dark gray. Humphrey et al. (1994) proposed the higher-level contributions of color to recognition, as visual agnosia patients also benefit from color information, and normal observers perform better with colors presented in their natural hues (Gegenfurtner and Rieger, 2000). Although this article did not explore the role of natural colors, it emphasized the effectiveness of high-brightness and low-saturation colors in this context. Chen et al. (2020) found that streets with a sense of place attachment play a mediating role in the emotional relationships of the elderly, aligning with the view of this article and highlighting the importance of emotional attachment in environmental satisfaction. In clinical records, Zhang et al. (2017) found that interface information complexity significantly impacts spatial positioning in patients with complex conditions, while this article’s proposed spatial visual information design model fully considers this inclusivity. Although the appropriate combination of color and graphics prompts spatial recognition in patients with visual–spatial impairment, this article recognizes that it may not be applicable in all situations. Park et al. (2023), Funayama et al. (2015), and Shirogane et al. (2017) support this view in their spatial cognition studies of feature cases.

The study of environmental adaptability for pathological characteristics may provide new ideas for achieving sustainable additional objectives (Megahed and Ghoneim, 2020), further revealing the potential of environmental design to aid rehabilitation. In the path to achieving environmental benefits for rehabilitation, “Stress Recovery Theory (SRT)” (Ulrich, 1983) and “Attention Restoration Theory (ART)” (Kaplan, 1995) are realized through distant viewing; the second stage of “Sensory Stimulation” rehabilitation theory proposed by Guo Tinghong is realized through the path from distant viewing to association. This research provides case evidence and strategic support for the third stage, “Behavioral Guidance,” proposed by the theory, but more supportive data is yet to be explored.

Based on ART, this paper explores the role of color and spatial design in aiding patients’ cognitive recovery. James (1986) laid the foundation for this theory by introducing the concepts of involuntary attention and directed attention. The former refers to attention that is naturally drawn without conscious effort, typically occurring in response to natural landscapes or novel stimuli. This allows individuals to restore psychological and cognitive function in a relaxed state. In contrast, directed attention requires sustained concentration, which may lead to mental fatigue. James (1984) argued that guiding patients into environments that trigger involuntary attention can alleviate attention fatigue and subsequently enhance overall cognitive performance.

Kaplan and Kaplan (1989) and Kaplan (1995) outlined several characteristics of environments conducive to effective attention restoration: (1) “Being Away” refers to a state in which individuals can temporarily escape daily stressors and cognitive demands, entering an environment distinctly different from their everyday life or work. This sense of detachment facilitates mental relaxation, helping individuals recover cognitive function; (2) “Extent” suggests that the environment should possess sufficient complexity and depth, offering ongoing opportunities for exploration. Rich details and expansive vistas can prolong the recovery of attention; (3) “Fascination” denotes an environment’s ability to naturally attract attention without the need for conscious focus. Elements such as natural scenery or artistic expressions can effectively reduce cognitive load and promote mental recovery; (4) “Compatibility” refers to a high level of alignment between an individual’s needs and the environment’s characteristics, ensuring seamless interaction without cognitive conflict; (5) “Legibility’ emphasizes the clarity and comprehensibility of the environment, allowing individuals to quickly grasp the spatial layout and direction, thus reducing the risk of disorientation.

These five environmental characteristics provide a framework for evaluating the effectiveness of public healthcare environment design in this study. By assigning distinct color schemes to different floors, the property of “Being Away” is reflected, helping patients dissociate from cognitively demanding everyday contexts and creating an experience distinct from routine activities, thereby alleviating cognitive stress (Zhang et al., 2024). The continuity of color coding enhances the environment’s “Extent” and “Legibility,” offering clear spatial cues that allow patients to maintain their sense of direction and spatial understanding during exploration, minimizing disorientation and uncertainty (Yesiltepe et al., 2021). Emotional connection, as a central factor influencing patients’ perception of the environment, surpasses the external function of traditional color aesthetics, fostering deep levels of “Fascination” and “Compatibility” (Gao and Zhang, 2020). Positive feedback from patients regarding the artificial tree decoration suggests that these emotional connections play a crucial role in the rehabilitation process, especially in therapeutic environments where such connections contribute to higher levels of patient satisfaction.

This paper offers a broad perspective on the interaction between environmental design and psychological recovery, with the following recommendations for advancing research in this field: (1) Investigate the complex interplay between color design and patients’ emotional memory, particularly in long-term care settings. For instance, explore how personalized decorative design can promote emotional recovery (Gao and Zhang, 2020); (2) Further research should examine the application of involuntary attention in healthcare spaces to understand how natural landscapes, art installations, and other elements can effectively reduce patients’ cognitive load (Marois et al., 2021); (3) The use of physiological data, such as heart rate variability or galvanic skin response, could quantify the actual impact of different color and spatial designs on attention recovery, providing more objective evidence to support design strategies (Bower et al., 2022); (4) A deeper exploration of cultural differences in patient responses to color and spatial design is needed, particularly in globalized healthcare settings, to develop adaptive design strategies tailored to diverse patient populations (Beck et al., 2020).

Methodologically, unlike Sung-Heui (2006), who directly used RGB colors as perceptual samples, this paper’s investigation using RGB values converted from the CIE color system is more scientifically valid (Gabbard et al., 2010). Existing research discusses the application differences between CIE and RGB colors, but most color perception surveys to date have not adequately considered spatial characteristics and commonly use RGB colors as survey samples. This approach overlooks the limitations of the RGB system in terms of visual perception authenticity, especially in complex environmental color reproduction. Chiang et al. (2018) have confirmed that the CIE color system has advantages over RGB colors in terms of spatial perception authenticity and consistency. This article, in extracting spatial color samples, fully considers the visual perception characteristics and environmental factors of colors, providing case support for the combined application of the CIE color system and the Analytic Hierarchy Process.

**Figure 9:**
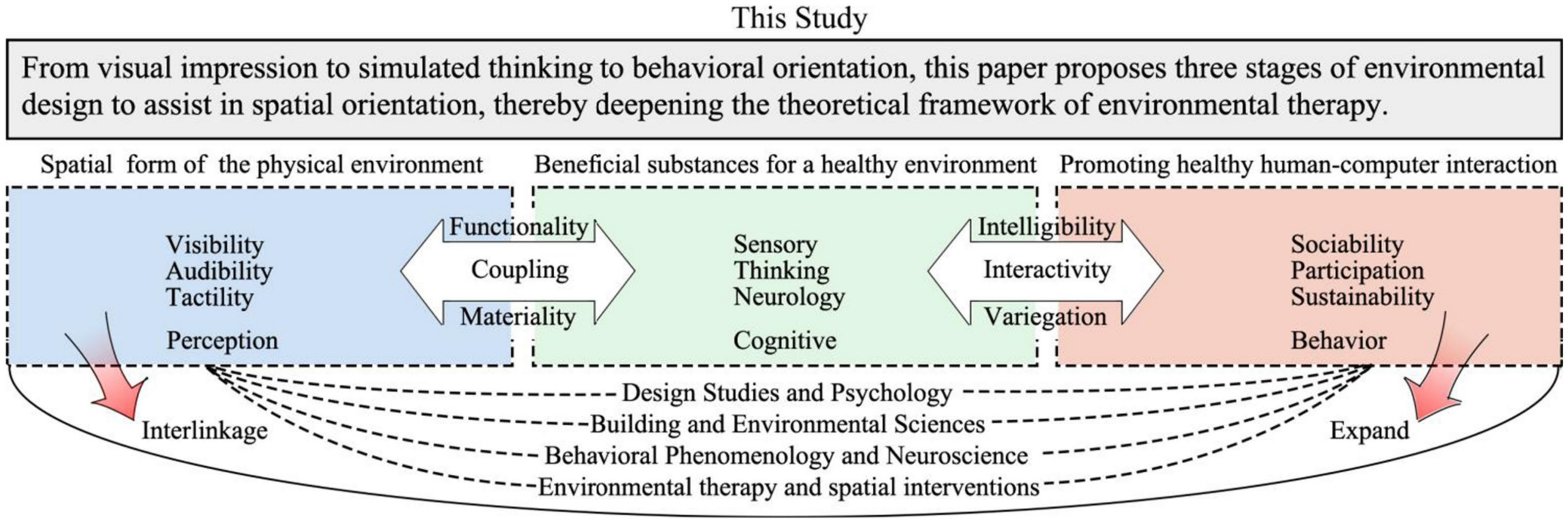
Academic contribution map.

Figure 9 in this article showcases its academic contributions. On the theoretical dimension scholars have explored “Environmental Intervention Therapy” from various perspectives, presenting a dynamic interplay across multiple disciplines such as design, architecture, and psychology. In the field of environmental design, three main aspects are emphasized: (1) In space perception, sensory stimuli such as visual, auditory, and tactile perceptions are often focused on; (2) In space cognition, visual impressions, thought stimulation and the mediating role of neural cognition in health promotion are common topics; (3) In behavior orientation, the focus is on the health sustainability of social participation behaviors. Methodologically, research on rehabilitative environment design has deeply expanded from spatial form to health materials to human-computer interaction. Based on the Analytic Hierarchy Process of CIE color space perception, this paper proposes design methods and models for assisting patients in spatial orientation, spanning from public environments to home-style therapeutic environments. It offers a comprehensive interpretation of the “interrelationship between visual–spatial impairment characteristics and environmental factors,” providing new perspectives for environmental therapy research.

Spatial intervention treatments based on patient pathological characteristics should prioritize individualized design needs while comprehensively considering the patient’s cognitive abilities, visual perception limitations, and behavioral patterns. For example, complex colors may lead to cognitive overload due to information overload (Jin et al., 2022), posing both a visual processing and cognitive load challenge. Additionally, issues such as spatial orientation, color contrast, and interface layout warrant attention—for instance, designing with consideration for the elderly’s sensitivity to colors (Pereira et al., 2021) or targeted designs for elderly patients with visual impairments (Tigwell, 2021). Balancing functionality and aesthetics is an essential aspect of environmental design work.

Despite the focus on spatial design as an entry point illustrating its potential to assist patient orientation, the technical realization remains challenging. At a broader level, universal design for the elderly still faces several challenges (Johnson and Finn, 2017; Min and Lee, 2020; Queirós et al., 2015; Merkel and Kucharski, 2019): (1) Front-end: A lack of integration between design concepts and practical applications leads to a disconnect between theory and practice, such as a shortage of flexible design strategies suitable for varying levels of cognitive ability. (2) Mid-stage: Investment in research and development far exceeds the execution capacity of actual projects, causing theoretical research to lag behind market demands. (3) Back-end: Regulatory strictness demands higher standards of environmental safety. (4) End-stage: The feasibility of design requires not only innovative thinking from designers but also mastery of human-computer interaction and psychology.

As research that integrates medicine, design, and psychology, this article has the following broader implications: (1) As evidence: The design strategies researched not only reflect the needs and challenges of individuals with visual impairments but also encapsulate most spatial design challenges, particularly applicable to medical and social welfare areas. This study provides deep insights into the development of assistive technologies, revealing key issues in fields such as color perception, interface adaptability, and behavioral analysis. (2) As a case: By using the research questions and findings proposed in this article, international scholars can validate the generality of these results through case comparisons and explore their applicability across different patient demographics. (3) As a paradigm: To comprehensively interpret the dynamic interplay between user needs and design responses, this article innovatively proposes methods and design models for color graphics to assist patient spatial orientation, providing a research paradigm for future environmental design.

This study interprets how the design of physical spatial elements can enhance and improve rehabilitation quality. Although it establishes connections between visual–spatial impairment and environmental factors and constructs a model for beneficial patient spatial orientation, the complexity of the condition’s onset mechanism leaves some space-driven factors unclear. This leads to new academic questions: (1) Beyond visual aspects, what other factors might influence patients’ spatial orientation, and how do these factors interact with the spatial environment; (2) Apart from spatial colors and graphics, can the redesign of other spatial elements improve patients’ spatial orientation abilities?

From the perspective of the pathological characteristics of visual impairments, the factors influencing patient spatial orientation and their interaction with other environmental elements can be explored in two main aspects: (1) Examining how changes in physical layout affect the ease of navigation and how these modifications reflect the usability of space. This involves investigating the specific impact of different spatial structures, such as the width of passageways, the placement of doors, and the configuration of accessibility facilities, on the mobility of patients. Further consideration is given to how design optimization can enhance the overall accessibility of the space, for instance, by increasing the clarity and recognizability of directional signs to reduce spatial confusion among patients; (2) Analyzing the psychological and sensory impacts of spatial design, and how these elements are influenced by both natural and artificial environmental conditions. Attention is paid to the roles of lighting, acoustics, and tactile feedback in shaping navigational behavior, as well as how adaptive technologies respond to new trends in environmental design. For example, exploring how different intensities of lighting and sound feedback affect the spatial perception of users with visual and auditory impairments, and how this information can be utilized to design more user-friendly interfaces. Additionally, the potential of multisensory integration technologies, such as integrated tactile maps and auditory navigation systems, to enhance the intuitiveness and efficiency of spatial orientation can be discussed, thereby providing more dynamic and inclusive environmental solutions.

The limitations of this paper are reflected in the slight lack of richness and diversity of the case studies. Firstly, the Guangzhou Yuexiu elderly service center follows a “daycare” model, and as such, its environmental conditions may differ from those of a nursing home that provides “full-day care.” Secondly, the epidemic reduced the number of respondents who could be reached during the visit, which had an impact on the survey’s findings on respondents’ preferences for different colous. Thirdly, although section 4.2 of this paper discusses the positive effects of sound color graphic design in supporting navigation tasks through a case study, behavioral intervention experiments are needed to validate the degree of effectiveness of the design strategy. Effective recommendations for studies like these include (1) increasing the number of case studies, such as increasing the percentage of interviewees in the pre-survey stage; and (2) enriching the diversity of cases, where the age, occupation, and gender of the patients can be distinguished for comparing the results; (3) Incorporate arousal theory (Nealis et al., 2016) into behavioral experiments (Li et al., 2024) in order to explore how color graphic design affects patients’ emotional responses and levels of cognitive arousal. A deeper case study can be conducted on the shape and color design of the pill bottle.

## 6 Conclusion

Findings: (1) mild cognitive impairment patients prefer bright, clean, and warm colors; (2) high brightness, low saturation, and distinct interface color designs increase spatial recognition for patients; (3) emotional attachment plays a key role in the elderly’s perception of environmental satisfaction; (4) complexity in interface information and scenes increases cognitive load, thereby reducing efficiency in locating and identifying spaces; (5) enhancing the visual impact of colors is not universally effective for all patients with visual–spatial impairment, but the universality of application can be increased through reasonable combinations and designs of colors and graphics.

From visual impression to cognitive stimulation to behavior orientation, this paper proposes a three-stage model and method for environmental design to assist in spatial orientation, exploring spatial design strategies. There is a correlation between the pathological characteristics of visual–spatial impairment (difficulty in spatial orientation, spatial neglect) and environmental factors. Assisting patients in spatial positioning through reasonable spatial interface design has potential, achievable through the combination of spatial color and graphic elements. As an interdisciplinary study, the model and results of this paper are theoretically feasible, as the reasoning process and methods are based on past successful experiences and cases. However, for practical application, COVID-19’s impact has restricted the team’s multiple attempts to visit and seek cooperation with relevant medical institutions. The emergence of innovative theories and methods requires support from extensive case data and experience, so the conclusions of this paper need more practical application and time for further validation.

The team’s future research focus will be to include other variables that influence spatial orientation as research subjects. The next step in the research plan is to seek cooperation with nursing homes or community service centers to apply this theory in design practice and contribute to building healthy living environments.

## Data Availability

The original contributions presented in the study are included in the article/supplementary material, further inquiries can be directed to the corresponding author.
